# Platelet signaling: a complex interplay between inhibitory and activatory networks

**DOI:** 10.1111/jth.13302

**Published:** 2016-04-09

**Authors:** A. P. Bye, A. J. Unsworth, J. M. Gibbins

**Affiliations:** ^1^ Institute of Cardiovascular and Metabolic ResearchSchool of Biological SciencesUniversity of ReadingReadingUK

**Keywords:** blood platelets, hemostasis, platelet activation, review, thrombosis

## Abstract

The role of platelets in hemostasis and thrombosis is dependent on a complex balance of activatory and inhibitory signaling pathways. Inhibitory signals released from the healthy vasculature suppress platelet activation in the absence of platelet receptor agonists. Activatory signals present at a site of injury initiate platelet activation and thrombus formation; subsequently, endogenous negative signaling regulators dampen activatory signals to control thrombus growth. Understanding the complex interplay between activatory and inhibitory signaling networks is an emerging challenge in the study of platelet biology, and necessitates a systematic approach to utilize experimental data effectively. In this review, we will explore the key points of platelet regulation and signaling that maintain platelets in a resting state, mediate activation to elicit thrombus formation, or provide negative feedback. Platelet signaling will be described in terms of key signaling molecules that are common to the pathways activated by platelet agonists and can be described as regulatory nodes for both positive and negative regulators.

## Introduction

The primary role of platelets lies in their ability to form aggregates when they encounter areas of blood vessel damage, allowing them to limit blood loss by forming a hemostatic clot. The capacity of platelets to aggregate also forms the basis of their role in disease, whereby platelet thrombus formation is triggered by an inappropriate stimulus such as the rupture of an atherosclerotic plaque. If the thrombus causes vessel occlusion in the heart or brain, this can result in a heart attack or stroke. The way in which platelets respond to their environment, often referred to as ‘platelet function’, defines their ability to fulfill their role in hemostasis and also their propensity to contribute towards disease. The environmental stimuli encountered by platelets are complex, as are the intracellular signaling pathways that regulate their responses to these stimuli.

**Figure 1:**
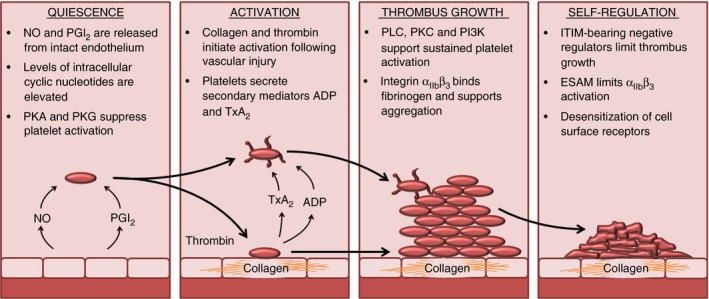
Stages of platelet activation and thrombus formation. Platelets in the circulation are kept in a quiescent state by nitric oxide (NO) and prostacyclin (prostaglandin I_2_; PGI_2_), which are released by the vascular endothelium. In platelets, NO and PGI_2_ increase the levels of cGMP and cAMP, and suppress platelet activity by the activation of protein kinase A (PKA) and protein kinase G (PKG). Following vessel injury, components of the subendothelial matrix are exposed, including collagen, which provides an adhesive surface for platelets to attach to and initiate signaling events and platelet activation. Local production of thrombin and secretion of secondary mediators also contribute to the initiation of platelet activation. Key components of platelet signaling pathways are activated, including phospholipase C (PLC), protein kinase C (PKC), and phosphatidylinositide‐3‐kinase (PI3K), supporting sustained platelet activation and thrombus formation through the initiation of cytoskeletal rearrangements, granule secretion, and activation of integrin α_II__b_β_3_. So as to limit thrombus growth and prevent the formation of occlusive thrombi, platelets contain self‐regulating negative feedback mechanisms that counteract positive signaling. These negative regulators include immunoreceptor tyrosine‐based inhibition motif (ITIM)‐containing receptors, endothelial cell‐selective adhesion molecule (ESAM), which negatively regulates integrin α_II__b_β_3_ activity, phosphatases that counteract phosphorylation‐dependent positive signaling, and receptor desensitization, which reduces the platelets’ response to secondary mediator signaling. TxA_2_, thromboxane A_2_.

To understand how platelets function within the vasculature, the three types of signals encountered by platelets and the signaling responses that they trigger must be considered simultaneously. Platelets encounter inhibitory signals that keep them in a quiescent state in the healthy vasculature, activatory signals at sites of vascular damage that rapidly trigger adhesion and aggregation, and negative regulatory signals that provide negative feedback once platelet activation is initiated and serve to limit thrombus formation (Fig. 1). There is a problem, however, with the conventional models used to understand platelet signaling. Platelets experience numerous extracellular signals at the same time and respond to complex combinations of primary and secondary activatory signals in addition to opposing inhibitory signals, yet we conceptualize such regulation as distinct linear pathways, each of which is triggered in response to a single agonist. This simplified approach is useful for designing experiments to delineate the order of events, but a more sophisticated approach is needed to incorporate multiple agonists and pathways, which themselves crosstalk, into a signaling network. Understanding these complex interacting signaling pathways as networks is a challenge that can only ultimately be met by systematic approaches that seek to model complex systems with many interacting parts. Systematic strategies that enable a holistic view of platelet function will be important to support the development of new antiplatelet therapies and new diagnostic methods, and successful prediction of hemostatic side effects for non‐platelet‐targeting drugs.

Many of the complex positive and negative platelet signaling pathways converge on key regulators of platelet signaling. By understanding how platelets regulate important signaling molecules such as phospholipase C (PLC), protein kinase C (PKC), and phosphatidylinositide‐3‐kinase (PI3K), which underpin critical events in platelet activation such as granule secretion and integrin α_IIb_β_3_ activation, we can understand the factors that define platelet function. In this review of platelet signaling pathways, we will refrain from describing signaling pathways as such, and instead focus on the impact of molecules that activate or inhibit platelet function on the core signaling processes that control platelet function, to begin to appreciate the interconnectedness of these systems.

## Regulators of platelet inhibition

Within the vasculature, platelets exist in a complex, flowing environment where both rheologic and hemodynamic factors force platelets into close proximity to the vessel wall. The signals generated by healthy, undamaged blood vessels promote quiescence, and allow platelets to circulate in a resting state 1. Without inhibitory signaling mechanisms, platelets would become activated even in the absence of activating signals. Circumstances in which these inhibitory signals are defective or activatory factors are exposed can lead to inappropriate platelet activation and cause ischemic heart disease or stroke 2.

The inhibitory signaling pathways are relatively few in number, but suppress several key nodes in the platelet signaling network that support activation. The primary platelet‐inhibiting signals generated by healthy, intact vascular endothelial cells are nitric oxide (NO) and prostacyclin (prostaglandin I_2_ [PGI_2_]), which regulate levels of intracellular cyclic nucleotides that suppress platelet activity by activating protein kinase G (PKG) and protein kinase A (PKA). 2.

### NO and PGI_2_

NO suppresses platelet activation by regulating intracellular levels of cGMP 3 via the activation of soluble guanyl cyclase, which regulates PKG activity. PGI_2_ binds to the prostaglandin receptor (IP receptor) on the platelet surface 4, and activates G‐protein α‐s‐coupled G‐protein‐coupled receptors (GPCRs). The active GTP‐bound form of Gs then binds to and activates adenylyl cyclase, and stimulates the production of cAMP from AMP 5, which in turn causes the activation of cAMP‐dependent PKA.

Many substrates of PKA and PKG are yet to be thoroughly characterized, but there is significant overlap in the well‐established targets. These targets include regulators of platelet activation such as Rap1b, which controls integrin α_IIb_β_3_ affinity, and is phosphorylated at Ser179 by PKA and PKG, causing it to be relocated away from the cell membrane, and downstream effectors of platelet activation 6. Cyclic nucleotides are also strong inhibitors of the release of Ca^2+^ into the cytosol, which underpins many events in platelet activation, including regulation of the Ca^2+^‐dependent Rap1 guanine nucleotide exchange factor (GEF), CALDAG‐GEFI 7. Phosphorylation of the inositol 1,4,5‐trisphosphate (IP_3_) receptor by PKA and PKG inhibits its function as a Ca^2+^ channel, and prevents Ca^2+^ release into the cytosol from the dense tubular system (DTS) 2. Targets specific to PKA include proteins involved in cell adhesion and spreading, such as Gα13 and GPIbβ. cGMP regulates cyclic nucleotide levels by activating phosphodiesterase (PDE)2A and PDE5A, which degrade cAMP and cGMP, respectively, but also inhibits PDE3A activity, reducing constitutive cAMP degradation 2.

Platelets from humans deficient in PKG have enhanced Ca^2+^ responses to agonists, indicating a role for cGMP‐mediated inhibition of Ca^2+^ release pathways 8. Mice lacking PKG have a prothrombotic phenotype and increased intravascular adhesion and aggregation following ischemia 9. Mice deficient in the IP receptor show enhanced platelet activation, highlighting the importance of Gs‐coupled receptors that regulate cAMP in suppressing platelet activation 10. Additionally, deletion of the IP receptor in atherosclerosis‐prone apolipoprotein E‐deficient mice significantly accelerates the development of atherosclerosis, which may be attributable, in part, to increased platelet reactivity 11.

### CD39

The endothelium and red blood cells can release several molecules, such as ADP and ATP, that are capable of stimulating platelets, and removal of these molecules is essential for the prevention of unwanted activation. CD39, an ectonucleotidase found at the platelet membrane and on endothelial cells, prevents platelet activation by hydrolyzing secreted ADP and ATP to AMP and adenosine 12. Adenosine activates the Gs‐coupled adenosine receptor, causing inhibition of platelets through elevation of cAMP 13.

### Junctional adhesion molecule A (JAM‐A)

JAM‐A, a member of the immunoglobulin superfamily of surface membrane proteins, has been identified in human platelets, and is thought to prevent platelet aggregation and thrombus formation in resting platelets through inactivation of integrin α_IIb_β_3_. In resting platelets, JAM‐A is phosphorylated and associates with integrin α_IIb_β_3_ to allow the recruitment of C‐terminal src kinase (Csk). Csk regulates autoinhibition of c‐Src through phosphorylation of the inhibitory Tyr529 site of c‐Src, thus preventing c‐Src‐dependent phosphorylation and activation of integrin α_IIb_β_3_ 14, 15. Upon strong agonist stimulation, JAM‐A is dephosphorylated, and this removes the brake on integrin α_IIb_β_3_ activation. As a consequence of this, mice deficient in JAM‐A show a hyper‐reactive platelet phenotype 15.

## Key regulators of activation

Following damage to the vascular endothelium, endogenous inhibitory signals are overcome, enabling platelets to react instantly to limit blood loss at sites of vascular injury. Platelets located close to the vessel wall within the blood flow come into contact with exposed extracellular matrix proteins such as collagen, and this, in combination with vascular shear forces, enables them to adhere to the site of injury and aggregate to form a thrombus 1. Platelets bind to the collagen‐containing adhesive surface through glycoprotein (GP)Ib–V–XI via von Willebrand factor (VWF) in the presence of high shear forces. Activation of the platelet receptors for collagen, integrin α_2_β_1_ and GPVI enables stable adhesion and initiates platelet signaling and activation 16. The release of secondary mediators such as ADP and thromboxane A_2_ (TxA_2_) amplifies the platelet response and subsequent activation of the coagulation pathway at the platelet surface. Exposure of phosphatidylserine (PS) on the surface of highly activated platelets enables assembly of the prothrombinase complex and the generation of thrombin. Thrombin is a potent platelet activator that can stimulate and enhance platelet aggregation and thrombus formation through the protease‐activated receptors (PARs) 17. Thrombin also stabilizes the growing thrombus by cleaving fibrinogen to form a fibrin mesh.

**Figure 2:**
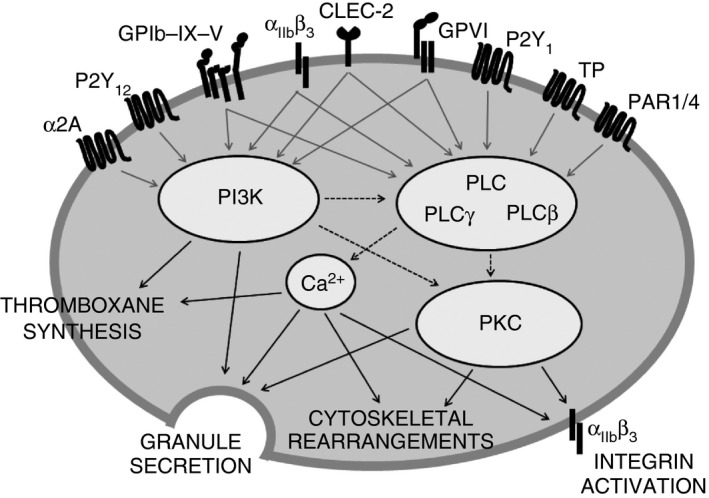
Phospholipase C (PLC), protein kinase C (PKC) and phosphatidylinositide‐3‐kinase (PI3K) are key mediators of platelet activation. All main activating platelet agonist receptors identified to date activate at least one of the following key activatory signaling mediators: PLC, PKC, or PI3K. These signaling nodes underlie several key processes required for platelet activation, including secretion of secondary mediators and activation of integrin α_II__b_β_3_, facilitating fibrinogen binding and platelet aggregation, and also cytoskeletal rearrangements, which enable platelet shape change and spreading. CLEC‐2, C‐type lectin receptor 2; GPVI, glycoprotein VI (collagen receptor); GPIb–IX–V, glycoprotein Ib–IX–V (von Willebrand factor receptor); PAR, protease‐activated receptor; P2Y_1/_P2Y_12_, ADP receptors; TP, thromboxane A_2_ receptor; α_II__b_β_3_, receptor for fibrinogen; α2A, adrenergic receptor.

These activating stimuli ultimately regulate a core set of signaling mediators that support activation. Three central mediator families of platelet activation are PLC, PKC, and PI3K, which are well characterized and underlie two of the critical events in platelet activation, i.e. secretion of secondary mediators and activation of integrin α_IIb_β_3_. These are by no means the only critical regulators of platelet function, but they represent key nodes in the complex network of platelet signaling that regulate many other important signaling elements (Fig. 2) 18, 19, 20, 21.

### PLC activation

Activation of PLC is a critical event in platelet activation, because it gives rise to two of the most important activatory signaling molecules in platelets. PLC family members catalyze the cleavage of phosphatidylinositol 4,5‐bisphosphate (PIP_2_) to generate diacylglycerol (DAG), which activates PKC, and IP_3_, which binds to IP_3_ receptors on the DTS of platelets to activate Ca^2+^ flux into the cytosol. Patients with low platelet PLC activity fail to respond normally to platelet agonists and even to Ca^2+^ ionophore, only achieving normal aggregation following addition of the direct PKC activator DiC_8_ 22. This is because both Ca^2+^ release and PKC activation are critical for integrin α_IIb_β_3_ activation and secretion. How and when the two main PLC isoforms in platelets, PLCγ2 and PLCβ, are activated therefore determines platelet activation.

PLCγ2 is activated downstream of immunoreceptor tyrosine‐based activation motif (ITAM)‐bearing (consensus sequence Yxx[L/I]x_6–12_Yxx[L/I]) adhesion receptors in platelets, i.e. GPVI‐FcRγ/FcγRIIA and C‐type lectin receptor 2 (CLEC‐2) (which bears a half‐ITAM motif [hemITAM]) 23. The pathway that activates PLCγ2 is complex, and involves assembly of a signaling protein complex, the linker of activated T cells (LAT) signalosome, around the scaffolding protein LAT, which is phosphorylated and activated by Syk downstream of Src family kinases 24. Activation of PLCγ2 requires binding of Slp76 to LAT via Gads, which enables Btk 25, 26 to phosphorylate PLCγ2 27. Clustering induced by collagen and fibrinogen binding to integrins α_2_β_1_ and α_IIb_β_3_, respectively, also stimulates PLCγ2 signaling, although for this LAT is not required 28. Owing to its role in the signaling cascade of receptors that mediate interactions stimulated by matrix proteins, PLCγ2 plays a significant role in initial platelet adhesion and thrombus formation. Genetic deletion of PLCγ2 in mice results in severely diminished thrombus formation following minor laser‐induced vessel injury, whereas its role was less significant following major injury, when thrombin generation plays a greater role 29.

PLCβ is activated by Gq‐coupled GPCRs such as P2Y_1_ (ADP receptor), TP (TxA_2_ receptor), and PAR1 and PAR4 (activated by thrombin). PLCβ is an important mediator of signaling evoked by the secreted, soluble secondary mediators ADP and TxA_2_, and therefore mediates positive feedback signaling and recruitment of platelets to growing thrombi. The consequences of preventing PLCβ activation via gene deletion of Gq in mice is profound, because it is the major route to Ca^2+^ release and PKC activation in platelets that are not in direct contact with matrix proteins 29.

### PKC activation

PKC constitutes a family of several different isoforms that play key regulatory roles in platelet intracellular signaling. Several isotypes/isoforms of PKC have been identified in platelets, and are divided into three different subtypes according to their structures and mechanisms of regulation: the conventional (classic) PKCs, i.e. PKCα, PKCβI/II, and PKCγ, which require both DAG and Ca^2+^ for full activation; the novel PKCs, i.e. PKCδ, PKCθ, PKCη, and PKCε (only in mice), which require DAG binding but are insensitive to Ca^2+^; and the atypical PKCs, i.e. PKCζ and PKCι/λ, which are insensitive to both DAG and Ca^2+^. Expression of some isoforms in platelets is controversial 19. Phosphorylation at key regulatory sites by phosphoinositide‐dependent kinase‐1 (PDK‐1) and autophosphorylation prime PKC for second messenger binding and activation 19. Studies that have utilized broad‐spectrum inhibitors and mice deficient in the individual isoforms of PKC have identified an overall positive role for the PKC family in the regulation of granule secretion, TxA_2_ synthesis, integrin activation, aggregation, and thrombus formation 19. Negative regulatory roles have also been identified, including roles in receptor desensitization and Ca^2+^ release, although some of the isoform‐specific roles remain controversial 19, 30, 31, 32. Substrates and targets of the PKC isoforms include components of the secretion machinery, such as SNAP23, syntaxin 4, and Munc18c, and cytoskeleton‐associated proteins, including pleckstrin, MARCKS, and vasodilator‐stimulated phosphoprotein (VASP), among other proteins that have been shown to be phosphorylated in a PKC‐dependent manner 19. These differences in function for the PKC family are attributed to different and distinct roles for the different isoforms in cell signaling and regulation, which could be attributable to different modes of regulation, different substrate specificities, and/or the association with distinct binding proteins and different subcellular localizations 33.

### PI3K activation

Appreciation of the role of class I PI3K in platelets as a promoter of sustained activation and stable thrombus formation has developed as its different functions have become understood. All class I PI3K isoforms phosphorylate PIP_2_ to generate phosphatidylinositol 3,4,5‐trisphosphate (PIP_3_), enabling signaling to or recruitment of proteins containing PH domains to the plasma membrane. This is believed to localize signaling kinases within close proximity of their downstream effectors. One such protein is the Ser/Thr kinase protein kinase B (Akt), which has numerous targets but is believed to exert some of its effects via inhibition of glycogen synthase kinase 3 (GSK3). Inhibition of GSK3 appears to potentiate responses to thrombin but not to GPVI agonists such as collagen 34. Following stimulation with thrombin, PKCα mediates early phosphorylation of GSK3, whereas phosphorylation by Akt occurs at later time points 35. Mitogen‐activated protein kinases such as extracellular signal‐regulated kinase (ERK) are also downstream effectors of PI3K, with an apparently minor role downstream of GPVI 36, 37. PI3K has an important role in the regulation of Btk and its substrate PLCγ2 by causing them to colocalize at the plasma membrane 38.

Mice deficient in each type I PI3K expressed in platelets have been generated and characterized 39. PI3Kδ is expressed at low levels, and genetic deletion or knock‐in of a catalytically non‐functional form had little measurable impact on platelet function. PI3Kγ is predominantly involved in signal transduction downstream of P2Y_12_, but plays a semi‐redundant role with PI3Kβ downstream of GPCRs. PI3Kβ is the dominant form downstream of GPVI, where it has a role in PLCγ2 activation and Ca^2+^ elevation 40. PI3Kα is believed to function cooperatively with PI3Kβ downstream of GPVI, where it also has a role in PLCγ2 activation 40, 41, possibly through the regulation of Btk. PI3K‐dependent activation of PDK‐1 may also contribute to the regulation of PKC activation.

## Key events during platelet activation

### Cytoskeletal rearrangement

Platelet activation is associated with major changes in the actin cytoskeleton that initially support shape change and later allow platelets to spread once they come into contact with adhesive surfaces such as exposed collagen or other platelets. Shape change occurs rapidly when platelets are stimulated; it results in the formation of pseudopodia that increase platelet surface area, and is dependent on Ca^2+^ elevation mediated by Gq or activation of G_13_, which couples to the small GTPase Rho. Rho mediates cytoskeletal reorganization through activation of myosin light chain kinase. Cytoskeletal reorganization enables relocalization of platelet granules and organelles to the center of the platelet, the transient formation of filopodia, and the sustained formation of lamellipodia, which enables secretion and spreading over the area of vessel damage 42. For spreading to occur, activation of either PLCβ or PLCγ, mobilization of intracellular Ca^2+^, the generation of phosphoinositides and activation of integrin α_IIb_β_3_ are required 43. Rapid reorganization of the actin cytoskeleton is characterized by a combination of uncapping, severing and nucleation of the actin filaments, and interaction with and activation of myosin II 44. Several regulators of these processes have been identified, including the Rho GTPases Rac, Cdc42 and RhoA, VASP, and PKC 19, 45, 46, 47.

### Secretion of secondary mediators

Secretion or release of secondary mediators is critical for the positive feedback that supports clot consolidation, but can also serve as the primary activating signal for platelets encountering an established and growing thrombus. The most prominent secondary mediators are ADP and TxA_2_. Like integrin α_IIb_β_3_ activation, the secretion pathways are dependent on several convergent signaling pathways activated by multiple receptors.

### Granule secretion

Platelets contain different types of granule, including the dense granules, which contain high concentrations of the secondary mediators ADP and 5‐hydroxytryptamine, and the α‐granules, which contain many coagulation and adhesive proteins. The mechanisms governing the secretion of both types of platelet granule appears to be largely shared, and involve assembly of a secretion complex containing soluble NSF‐associated attachment protein receptor (SNARE) proteins such as the vesicle‐associated membrane proteins (VAMPs) 48, and SNARE regulators such as Munc13‐4 49. Ca^2+^ has a critical role in granule secretion in all cell types, but the precise mechanism of Ca^2+^‐mediated granule secretion in platelets is unclear. It is believed that the Ca^2+^‐sensing protein calmodulin mediates phosphorylation of myosin light chain 50, leading to interaction with VAMP and the exocytotic cell machinery, causing granule secretion 51. Slp1 inhibits dense granule secretion until the inhibition is relieved by binding of Rap1GAP2, although the role of Ca^2+^ in the process is unclear 52.

The DAG mimetic and PKC activator phorbol 12‐myristate 13‐acetate can induce granule secretion in the absence of intracellular Ca^2+^ elevation 53. Although it has long been understood that PKCs have a net positive regulatory role in granule secretion, the precise roles of the different isoforms are difficult to dissect. Negative roles for both PKCδ and PKCθ following stimulation by GPVI agonists have, however, been described 19. Many key regulators of granule secretion, such as Munc18c, syntaxin‐4, and SNAP23, are phosphorylated, and therefore potentially regulated, by PKCs 54, 55, 56.

As a consequence of the complex crosstalk between pathways, the secreted secondary mediators have a role in potentiating secretion, creating an unintuitive, cyclical relationship. For example, following GPVI activation, P2Y_12_ responds to secreted ADP by potentiating granule secretion via activation of Rac1, leading to an increase in ADP secretion and further activation of P2Y_12_ and P2Y_1_ 57.

### TxA_2_ synthesis

TxA_2_ is synthesized *de novo* upon platelet activation. Synthesis is mediated by a cascade of enzymes, including cyclooxygenase‐1, the target of aspirin, but it is regulated at the level of liberation of arachidonic acid from membranes by phospholipase A_2_. This enzyme is activated by elevated Ca^2+^, which induces translocation to the plasma membrane and phosphorylation by the stress kinase P38 58 and ERK1/2 59. As is the case in granule secretion, P2Y_12_ has a regulatory role in TxA_2_ synthesis, whereby stimulation of platelets with ADP causes PI3K‐dependent TxA_2_ generation, which potentiates Ca^2+^ signaling 60.

### Novel secretion pathways

A novel platelet secretion mechanism has recently been reported whereby ATP is secreted from pannexin hemichannels to activate P2X_1_, the only ligand‐gated Ca^2+^ channel in platelets, causing influx of Ca^2+^. This enhances activation stimulated by low concentrations of GPVI agonists 61.

### Integrin activation

In many ways, integrin α_IIb_β_3_ activation represents the central point of platelet signaling pathways, as the majority of inhibitory and activatory signaling pathways contribute towards its regulation in some way. The activation of integrin α_IIb_β_3_ is the culmination of a chain of signaling events that induce a conformational change enabling high‐affinity binding of fibrinogen and VWF. Without the capacity to bind fibrinogen, platelets are unable to adhere to each other and form aggregates. The binding partners that are assembled around integrin α_IIb_β_3_ and support activation and association with the actin cytoskeleton include talin 62 and kindlin 63. The small GTPase Rap1 is an important regulator of integrin α_IIb_β_3_ activation 57, 64, and appears to be the integrating point of many platelet‐activating signals. Elevation of Ca^2+^ modulates Rap1 activation via the highly expressed platelet GEF CalDAG‐GEFI 65, which binds Ca^2+^ via EF‐hand domains to become activated 66. Activation of PI3K is another central event in sustained platelet activation, and this too modulates Rap1 67. PI3K has recently been shown to act by inhibiting the GTPase‐activating protein RASA3, which negatively regulates Rap1 activation 68. This mechanism might explain why activators of PI3K, such as the ADP receptor P2Y_12_, are unable to elicit integrin α_IIb_β_3_ activation unless positive regulation via Ca^2+^ elevation or PKC occurs simultaneously. A study using genetic reconstitution of the integrin α_IIb_β_3_ activation pathway in cell lines, RIAM, indicated that the Rap1b effector was a critical mediator of integrin activation in platelets, linking the GTPase to integrin α_IIb_β_3_ 69. However, a recent study has demonstrated that the adaptor molecule might not have a critical role in platelets, as platelet‐specific RIAM deficiency in mice was not accompanied by a platelet functional defect 70.

Advanced microscopy techniques are now revealing the spatial and temporal regulation of integrin activation, and have demonstrated that knowledge of the signaling cascades that support activation of key platelet receptors such as integrin α_IIb_β_3_ is only part of the story. Coordination of the cytoskeleton with clustering of adhesive receptors and mediators of activation underlies the ability of platelets to form stable aggregates under shear stress 71.

## Negative feedback and self‐regulation

Following initiation of platelet activation and thrombus formation, negative signaling mechanisms restrain activation to ensure that platelet aggregation does not progress out of control, thus preventing excessive thrombus formation.

The pathways that contribute to negative regulation have been studied less extensively than those that mediate platelet activation, and consequently have not been fully characterized. Negative regulators, such as immunoreceptor tyrosine‐based inhibition motif (ITIM)‐containing receptors, are thought to reduce the activation of critical players such as PLC, PI3K, and integrin α_IIb_β_3_ 72. Other endogenous inhibitory mechanisms include endothelial cell‐selective adhesion molecule (ESAM), Wnt–β‐catenin, and semaphorin 3A (Sema3A), which negatively regulate integrin α_IIb_β_3_ activity, phosphatases that limit phosphorylation‐dependent mechanisms, receptor desensitization, which limits the response to secondary mediator signaling, and intracellular nuclear receptors with different mechanisms of action 73, 74.

### ITIM signaling

ITIM‐containing proteins are predominantly associated with negative regulation of platelet signaling and activation. The ITIM consensus sequence (L/I/V/S‐x‐Y‐x‐x‐L/V) in the cytoplasmic tail has been identified in several proteins that are expressed in platelets, including platelet endothelial cell adhesion molecule‐1 (PECAM‐1) 75, 76, carcinoembryonic antigen cell adhesion molecule‐1 (CEACAM‐1) 77, carcinoembryonic antigen cell adhesion molecule‐2 (CEACAM‐2) 78, G6b‐B 79, 80, and LILRB2/paired immunoglobulin‐like receptor B (PIRB) 81, all of which negatively regulate platelet activation. PIRB is activated by binding of its endogenous ligand, ANGPTL2, which is secreted by platelets 81. In contrast, PECAM‐1 appears to be activated by receptor clustering that is stimulated by homophilic interactions 82. For other ITIM receptors, it is not clear which type of interaction might trigger activation, but their function can be inferred from the phenotypes of mice with genetically induced deficiencies for different ITIM‐containing receptors. Activation of ITIM receptors evokes src family kinase‐dependent phosphorylation of the ITIM tyrosine residues, which enables the recruitment of negative regulators of platelet activation, including phosphatases such as SHP1/SHP2 83 and SHIP1/SHIP2, to the receptor 84. Binding to the ITIM brings these proteins in close proximity to their substrates. The resulting inactivation of tyrosine kinases such as Syk, inactivation of the LAT signalosome and PLCγ2 and inhibition of PIP_3_ leads to inactivation of PI3K and Akt, and inhibition of downstream signaling pathways 85. Recruitment to the ITIM can also result in the relocalization of molecules away from other signaling partners, preventing further transmission of positive signals such as that observed with PECAM‐1, which associates with PI3K and sequesters it away from LAT, preventing its activation and downstream signaling 85.

ITIM‐containing proteins were generally considered to constitute the off ‘switch’ that counteracts the positive signaling initiated by ITAM‐bearing receptors, such as GPVI‐FcRγ/FcγRIIA, and CLEC‐2 (which contains a hemITAM). However, studies on PECAM‐1 75, 76, 86, G6b‐B 79, 80 and LILRB2/PIRB 81 have identified inhibitory regulatory roles following stimulation by GPCR agonists and in the regulation of integrin α_IIb_β_3_ function, suggesting that their role is not solely limited to inhibition of ITAM signaling. In support of an inhibitory role in the regulation and limitation of platelet activation, studies of thrombus formation in PECAM‐1 87, CEACAM‐1 77, CEACAM‐2 78 or LILRB2/PIRB 81 receptor‐deficient mice have shown that the mice have hyper‐reactive platelets, increased thrombus size and increased stability of thrombi as compared with wild‐type controls.

Interestingly, despite the negative roles of PECAM‐1 and CEACAM‐1 in the regulation of platelet activity, studies using either PECAM‐1^−/−^ or CEACAM‐1^−/−^ mouse platelets show impaired outside‐in signaling, as spreading and adhesion on fibrinogen, clot retraction and phosphorylation of focal adhesion kinase are reduced in comparison with wild‐type platelets 88, 89. This indicates positive roles for both PECAM‐1 and CEACAM‐1 in the regulation of integrin α_IIb_β_3_ signaling following fibrinogen binding.

Human studies have revealed that the expression levels of PECAM‐1 on the surface of human platelets correlates negatively with platelet reactivity to CRP‐XL and ADP. The contribution of PECAM‐1 expression levels as a determinant of platelet reactivity is around half that of GPVI levels, demonstrating the importance of both activatory and inhibitory pathways in controlling responsiveness. 76.

### Other negative regulators

ESAM, which is usually contained in the α‐granules, is translocated to the cell surface following platelet activation 90, and is suggested to be involved in the negative regulation of integrin α_IIb_β_3_ outside‐in signaling, as ESAM‐deficient mouse platelets show increased aggregation in response to GPCR agonists, inhibition of clot retraction, increased thrombus formation *in vivo*, and reduced tail bleeding 91. The mechanism by which ESAM functions is currently unknown, although interaction with the scaffold protein NHERF‐1 highlights the possible regulation of several proteins, including GPCRs, G‐proteins, PLCβ, and components of the cytoskeleton.

The neurophillin‐1–plexin A receptor complex, which has been identified in platelets 92, is activated by Sema3A, which is secreted from endothelial cells 93 and negatively regulates platelet function through regulation of integrin α_IIb_β_3_. Integrin activation, aggregation, adhesion and spreading are all impaired following Sema3A binding. The exact mechanisms by which Sema3A binding inhibits platelet function have yet to be fully elucidated; however, inhibition of the GTPase Rac1 could play a role 92.

Wnt3a is a GP that has been found to be released from TRAP‐stimulated platelets, enabling platelets to self‐regulate and limit activation 94. In platelets, Wnt3a is thought to activate the canonical Wnt–β‐catenin signaling pathway, as several components of this pathway have been identified in platelets 94. Wnt binding leads to the inhibition of platelet adhesion and shape change, dense granule secretion, integrin α_IIb_β_3_ activation, and aggregation, possibly through regulation of the activity of small GTPases, including Rap1, Cdc42, Rac1, and RhoA 95.

Intracellular nuclear receptors have recently been identified in platelets and found to have a role in platelet inhibition. Usually associated with genomic regulation of transcription, these receptors have also been shown to have non‐genomic regulatory roles. Several intracellular nuclear receptors have been identified and characterized in human platelets, including peroxisome proliferator‐activating receptor (PPAR)α, PPARβ/δ, PPARγ, retinoid X receptor (RXR), liver X receptor (LXR), and glucocorticoid receptor 96, 97, 98, 99, 100. Treatment of platelets with ligands for these receptors results in inhibition of platelet aggregation in response to several platelet agonists, supporting previously published cardioprotective effects of these treatments 96, 97, 98, 99, 100, 101, 102. The different nuclear receptors can interact with each other to form heterodimers, although, to date, little is known about the interactions between these receptors in platelets. There is some overlap in the mechanisms by which they inhibit platelet activation, highlighting possible points of receptor interaction. Ligands for PPARα and PPARβ/δ appear to elicit their inhibitory effects through regulation of cellular cAMP levels 103, whereas PPARγ and LXR ligands alter association of the receptor with components of GPVI and integrin signaling pathways such as Syk, LAT, and PLCγ2 96, 98.

Protein modification by phosphorylation of tyrosine or serine and threonine residues is a key reversible mechanism of signal transduction. Dephosphorylation by phosphatases enables bidirectional regulation of signal transduction and platelet function. Phosphatases, including SHP1/SHP2, SHIP1/SHIP2, protein phosphatase (PP)2, phosphatase and tensin homolog (PTEN), and T‐cell ubiquitin ligand‐2 (TULA2), are integral mediators of several negative signaling mechanisms in platelets preventing key platelet processes such as mobilization of intracellular Ca^2+^, granule secretion, and integrin activation. SHP2 has well‐established roles in the negative regulation of platelet signaling downstream of both GPCRs and GPVI, and is a key mediator of ITIM receptor signaling downstream of PECAM‐1 and CEACAM‐1, negatively regulating proximal signaling events, leading to reduced PLC activation and therefore reduced mobilization of Ca^2+^ 73. TULA2 dephosphorylates and inactivates Syk, preventing signaling that has been shown in mathematical modeling studies to be critical in determining the rate of GPVI‐mediated platelet activation 104. The inositol phosphatases PTEN and SHIP1 play key roles in altering the phosphoinositide cycle and antagonizing PI3K function 105. The role of the serine/threonine kinase phosphatase PP2A in the negative regulation of integrin α_IIb_β_3_ function and signaling has been attributed to the dephosphorylation of PKCζ, and protein‐tyrosine phosphatase 1B, which subsequently reduces Src phosphorylation and activation 106, 107, 108. Although several phosphatases are associated with negative regulation of platelet function, it is important to note that many positive regulatory functions for phosphatases, e.g. CD148, have also been identified in platelets 73.

Receptor desensitization enables platelets to regulate their responsiveness to platelet agonists. The process of receptor desensitization includes inactivation via phosphorylation events, and internalization, which removes receptors from the plasma membrane. As previously described, ADP is a critical secondary mediator of platelet activation, but the receptors for ADP are rapidly desensitized following exposure to ADP, resulting in a subsequently reduced agonist response 30, 74. This diminished response is attributed to desensitization of both the P2Y_1_ and P2Y_12_ receptors via agonist‐mediated internalization through two different protein kinase‐mediated pathways, with P2Y_1_ desensitization being mediated by both classic and novel isoforms of PKC, and P2Y_12_ desensitization being mediated by GPCR kinase and the novel PKC isoforms 30, 74. In contrast, the PARs have been shown, following platelet activation by thrombin or the PAR1 peptide agonist SFLLRN, to be internalized or shed into membrane microparticles, resulting in a decrease in the number of receptors detected at the platelet surface. This decrease is thought to underlie the inability of platelets to recover their responsiveness to PAR agonists 109.

## Novel approaches to platelet signaling and future directions

**Figure 3:**
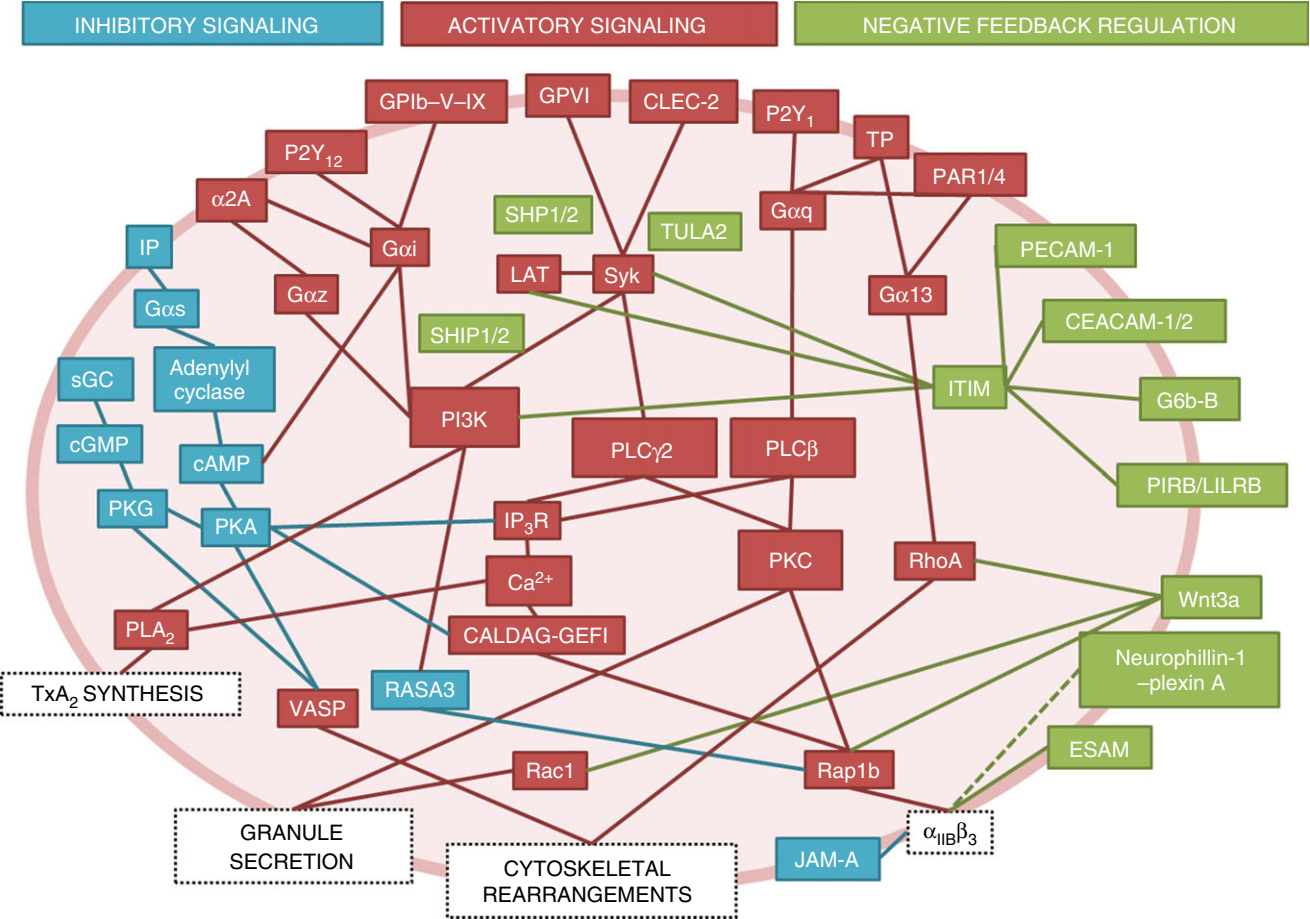
Complexity of platelet signaling networks. Platelet signaling models frequently describe signaling pathways activated by individual agonists; however, platelet signaling *in vivo* is highly complex, and involves simultaneous activation by multiple agonists and negative regulators, which form a complex signaling network. Several key signaling molecules, i.e. phospholipase C (PLC), protein kinase C (PKC), and phosphatidylinositide‐3‐kinase (PI3K), are common between the different pathways and form key nodes of platelet regulation. Blue boxes and lines represent mediators of inhibitory signaling that act to suppress platelet function in the absence of platelet activators in the healthy endothelium. Red boxes and lines represent mediators of activatory signaling following platelet activation by platelet agonists. Green boxes and lines represent mediators of negative feedback and inhibitory signaling that act to limit platelet activation following stimulation by platelet agonists. CALDAG‐GEFI, Ca^2+^‐dependent Rap1 guanine nucleotide exchange factor; CEACAM‐1/2, carcinoembryonic antigen cell adhesion molecule‐1/2; CLEC‐2, C‐type lectin receptor 2; ESAM, endothelial cell‐selective adhesion molecule; GP, glycoprotein; IP, prostaglandin receptor; IP_3_ R, inositol trisphosphate receptor; ITIM, immunoreceptor tyrosine‐based inhibition motif; JAM‐A, junctional adhesion molecule A; LAT, linker of activated T cells; PAR, protease‐activated receptor; PECAM‐1, platelet endothelial cell adhesion molecule‐1; PIRB, paired immunoglobulin‐like receptor B; PKA, protein kinase A; PKG, protein kinase G; PLA_2_, phospholipase A_2_; sGC, soluble guanyl cyclase; TULA2, T‐cell ubiquitin ligand‐2; TxA_2_, thromboxane A_2_; VASP, vasodilator‐stimulated phosphoprotein; α2A, adrenergic receptor.

The understanding of platelet signaling has increased exponentially in recent decades, and it is now clear that the signaling that underpins platelet function is best described as a heavily interlinked network rather than as linear pathways (Fig. 3). The vast amount of data describing the components within this network presents new challenges regarding the best way to interpret and transform this information into improved diagnostic and therapeutic strategies. Progressive approaches to platelet function screening have been developed that utilize standardized assay plate technology, bringing the high‐throughput methodologies of the drug discovery industry into the clinical setting 110. The ability to easily test many parameters simultaneously may allow positive and negative pathways to be investigated simultaneously and enable more of the platelet signaling network to be considered during the diagnosis of platelet disorders. Systematic measurement and analysis of platelet function under flow is also a promising avenue of development, and provides a sensitive diagnostic approach under more physiologic conditions and incorporates many more aspects of the platelet signaling network into the diagnosis of platelet disorders 111. The use of both of these strategies in combination with genetic mutant models, patient studies and inhibitor screening would give us a thorough insight into the roles of particular molecules in the regulation of platelet activity as a whole.

Systems approaches that utilize mathematical modeling are increasingly being applied to help in our understanding of platelet biology. These can take two distinct forms: those using a top‐down approach, whereby the properties of relatively intact and complex systems are modeled; and those using a reductionist approach, whereby relatively few aspects of platelet signaling or function are modeled, but in greater detail. For example, the top‐down approach has revealed important details of how platelet thrombi are organized into a highly activated, dense ‘core’ of PS‐exposing platelets surrounded by a loosely packed and partially activated ‘shell’ 112, 113, 114. Reductionist models, in contrast, often focus on Ca^2+^ signaling, as it is easy to measure with high temporal resolution and to quantify, and is a point of signal integration downstream of many platelet pathways. Ca^2+^ models have proved to be useful in understanding how platelets organize important functional events, such as PS exposure, in subpopulations 115. Modeling of the processes that regulate cytosolic Ca^2+^ levels has provided insights into the mechanism of store‐operated Ca^2+^ entry and release from the DTS via IP_3_ receptors 116. Mathematical modeling has aided the understanding of kinase regulation downstream of GPVI and in particular the important role that negative regulation by phosphatases, such as TULA2, play in modulating signal transduction 104.

Future innovations will be required to integrate pathways into networks and to develop top‐down and reductionist models until they are able to meet in the middle. Such complex models will need to be validated by the use of data from many different platelet donors, in order to reflect the variability in platelet responsiveness that is present within the human population. Such modeling of platelet signaling networks will prove to be an important future direction for the field, as it will be a requirement for understanding how combinations of regulatory factors contribute collectively to platelet function and predicting how changes within specific parts of the network alter platelet reactivity or levels of activation. This knowledge will aid in the selection of proteins (individually or in combination) representing promising new antithrombotic targets, and will help in understanding the defects associated with an increased risk of thrombosis.

## Disclosure of Conflict of Interests

The authors state that they have no conflict of interest.
